# Factors associated with violence against women following the COVID-19 lockdown in France: Results from a prospective online survey

**DOI:** 10.1371/journal.pone.0257193

**Published:** 2021-09-10

**Authors:** William Peraud, Bruno Quintard, Aymery Constant

**Affiliations:** 1 Department of Psychology, Bordeaux University, Bordeaux, France; 2 Department of Social and Behavioral Sciences, EHESP School of Public Health, Rennes, France; Qazvin University of Medical Sciences, ISLAMIC REPUBLIC OF IRAN

## Abstract

**Background:**

The aim of this research was to investigate the impact of the first COVID-19 lockdown (March 17^th^—May 11^th^ 2020) on violence against women in France.

**Methods:**

A prospective survey was conducted online between April 2th 2020 and July 5^th^ 2020. Female respondents were recruited from social media networks using the snowball sampling method. Data were collected three times: during (2–19 April) and at the end (11–25 May) of the first lockdown, and following the first lockdown (20 June– 05 July). Sociodemographic variables, lockdown living conditions, financial impact of COVID, and history of psychiatric disorder were evaluated, together with changes in psychological distress over the lockdown period, and the risk of being assaulted post lockdown.

**Results:**

Psychological distress was elevated and remained stable for most of the 1538 female respondents during lockdown. More than 7% of women were affected by physical or sexual violence post lockdown. Unwanted sexual contact accounted for the majority of abuse, but physical and sexual assault were also prevalent. The risk of being abused was higher for participants who had changed anxiety/insomnia symptoms over the lockdown period, and a history of abuse.

**Discussion:**

Women who experienced changes in anxiety/insomnia symptoms during the COVID-19 lockdown were at higher risk than others of being assaulted post lockdown, especially when they were already socially vulnerable. While social and psychological factors accounting for these changes warrant further investigation, communication and preventive measures during pandemics should include initiatives tailored to women more vulnerable to violence.

## Introduction

The coronavirus (COVID-19) pandemic has had worldwide impact with more than ten million cases and more than 500,000 deaths by July 1th 2020 [1]. Several measures were implemented to prevent further spread of the disease in the early stages of the pandemic. Lockdown, the restriction of individuals to their homes, was one of the measures enforced in many countries, including France. Lockdown and physical distancing helped control the first wave of the COVID-19 pandemic, but also had significant negative consequences for individuals’ mental and physical health [2, 3], especially for people with high levels of COVID-19 anxiety [4]. Early in 2020, the World Health Organization (WHO) expressed concerns about the effects of COVID-19 on psychological wellbeing [5]. Essential workers, particularly health care professionals, were reported to be at increased risk of negative psychological effects [6–9]. Studies have consistently reported increased rates of COVID-related psychological distress amongst the population, particularly symptoms of anxiety, depression and insomnia [10–13].

Of particular concern, mental health disorders constitute a significant vulnerability factor for violence against women [14, 15], which was rapidly emerging as a major public health problem in the midst of the quarantine [16–20]. Reports of increasing rates of domestic violence against women during the COVID-19 pandemic began to surface around the world [21]. France registered a 30% increase in domestic violence reports, Brazil estimated domestic violence reports to have increased by 40–50%, and Italy indicated that reports of domestic violence are on the rise [22]. The growing global trend of increasing reports of domestic violence cases is likely to continue throughout the pandemic and may only represent the “tip of the iceberg” as many victims still find themselves trapped with the perpetrator and unable to report the abuse [21].

Better knowledge of the manner in which the COVID-19 lockdown affected violence against women may contribute to designing prevention initiatives during and after pandemics. But rigorous studies examining the relationship between violence against women and the COVID-19 pandemic are scarce; most of the articles are commentaries, letters, editorials, and most of the published data derives from social media, internet, anecdotal evidence and helplines reports [19]. The aim of this research was to investigate the influence of the first lockdown (March 17^th^—May 11^th^ 2020) on violence against women in France. Specific objectives were to 1) prospectively assess changes in psychological distress during the first lockdown among women from the general French population, and 2) investigate the relationship between psychological distress during lockdown and the risk of being the victim of violence post lock-down.

## Materials and methods

### Participants and procedures

**Fig 1:**
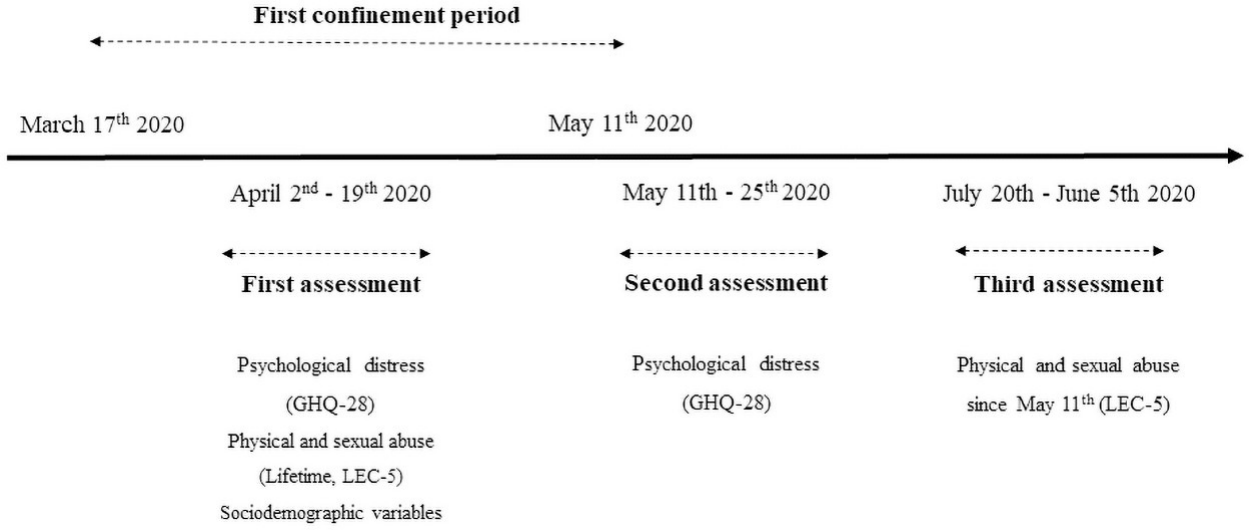
Prospective study design. Data were collected at three time points: during (2–19 April) the first lockdown and at the end (11–25 May), and post lockdown (20 June– 05 July). Note: GHQ-28: General Health Questionnaire 28 items; LEC-5: Life events checklist for DSM5.

A prospective online survey was conducted in the adult general French population between April 2nd 2020 and July 5^th^ 2020 (Fig 1). Respondents were recruited from social networks (Facebook, Instagram, LinkedIn) using the snowball sampling method. Inclusion criteria were female sex, age ≥ 18 years, able to communicate fluently in French, and consenting to participate to the study. Exclusion criteria were male sex; age < 18 years; inability to communicate fluently in French and refusal of participation. Data were collected at three time points: during (2–19 April) the first lockdown and at the end (11–25 May), and post lockdown (20 June– 05 July). At the first assessment, respondents were asked to provide their email addresses together with a personal identity code (ID code). Data from the three assessments were matched using the ID code, as email addresses and other data were recorded in separate databases in a locked computer. Only respondents who completed the three assessment were included in the analyses. The research protocol met the General Data Protection Regulation criteria, and was approved by the Institutional Review Board of the University of Bordeaux. Participants were informed of the topics to be discussed, and the manner and form in which data were collected and confidentiality maintained. Written/oral consent was not obtained as the data were analyzed anonymously.

### Measures

Psychological distress was assessed using the French version of General Health Questionnaire [23]. The 28-item version (GHQ-28) identifies four dimensions with excellent internal consistency, as assessed by the Cronbach alpha: somatic symptoms (α = 0.75), anxiety and insomnia (α = 0.80), social dysfunction (α = 0.90) and severe depression (α = 0.90) [24]. Each dimension is composed of seven items, each with four response modalities referring to the frequency of the difficulty (not at all, not more than usual, a little more than usual and much more than usual). Scores of 1 are given to answers that reflect an increase of difficulties. For example, for the item “Have you recently lost much sleep over worry?”, the respondent could answer “not at all” (scored 0), “not more than usual” (scored 0), “a little more than usual” (scored 1) or “much more than usual” (scored 1). Respondents with a global score greater than or equal to 6 are considered to present psychological distress. In the first wave, the temporal reference was the beginning of the lockdown. For the second and third waves, the temporality was based on the past month.

Violence against women was assessed using the French version of the Life Event Checklist for DSM-5 [25]. This scale lists 17 categories of traumatic events. Participants indicated if they had experienced or witnessed each type of event. In the present study, the assessment of traumatic events was limited to sexual or physical assault they had experienced over their lifetime and in the last month, namely: unwanted sexual contact, physical assault, sexual assault and assault with a weapon.

Sociodemographic and environmental variables included age (in years), level of education (University degree; high school graduate; high school undergraduate), occupational status (student; inactive; active), psychiatric history (none; depression; other) and type of companionship during the lockdown (friends/roommate; family; partner; alone). Using 5-point scales, participants were asked to rate the negative impact of COVID-19 outbreak on their financial situation (Very high = 5; High = 4; Moderate = 3; Low = 2; None/very low = 1) and their satisfaction with their social relationships during the lockdown (Excellent = 5; Good = 4; Moderate = 3; Poor = 2; Very poor = 1).

### Data analysis

Categorical data are expressed as numbers (N) and percentages (%) and compared with chi-square test, while numerical data were expressed as means ± standard deviations and compared with one-way analysis of variance. Internal consistency reliability was assessed by computing Cronbach’s α coefficient, considered satisfactory if higher than or equal to≥ 0.70. GHQ subscales scores were considered as having increased (or decreased) over the lockdown period if they have increased (or decreased) by at least 2 points between the first and the second assessment, and “unchanged” otherwise. Since our study outcome was a count variable (number of abuse/assault categories experienced post lockdown), we used generalized linear Poisson regression models to estimate the rate ratios (RRs) of violence against women as a function of sociodemographic variables and changes in psychological distress over the lockdown period. Estimates in univariate analysis (model 1) were expressed as Rate Ratios with 95% confidence intervals (RR [95% CI]). Significant estimates (*P*<0.10) from model 1 were input in a multivariate model (model 2). Statistical analyses were performed using the SPSS statistical package, version 19 (SPSS, Chicago, Illinois, United States).

## Results

Of the 7176 women who responded to the first online survey and provided their email addresses, 3408 (47.5%) completed the second assessment and 1770 (24.7%) completed the third assessment. Of these, 232 participants were removed from analyses because of missing sociodemographic data. Comparisons revealed that women with a university degree, a psychiatric history, a higher age and lower GHQ scores during the lockdown were less frequently lost to follow-up than others (Table 1).

**Table 1:** Sociodemographic of female respondents according to study participation and inclusion (N = 1538).

		Excluded	Included	*P*-value †
Variables		N = 5638	N = 1538	
Mean age in years (SD)		34.5 (12.2)	35.5 (12.1)	0.004
Occupational status	Student	1015 (18.0)	310 (20.2)	0.066
	Inactive	665 (11.8)	195 (12.7)	
	Active	3958 (70.2)	1033 (67.2)	
Education level	University degree	4136 (73.4)	1271 (82.6)	<0.001
	High school graduate	1180 (20.9)	209 (13.6)	
	High school undergraduate	322 (5.7)	58 (3.8)	
Psychiatric history	Others disorders	287 (5.1)	101 (6.6)	0.010
	Depression	435 (7.7)	141 (9.2)	
	None	4916 (87.2)	1296 (84.3)	
Lockdown Companionship	With Friends/roommate	718 (12.7)	194 (12.6)	0.178
	With Family	2835 (50.3)	729 (47.4)	
	With a Partner	1391 (24.7)	413 (26.9)	
	Alone	694 (12.3)	202 (13.1)	
Social relationship satisfaction	Excellent	1802 (36.7)	491 (36.8)	0.717
	Good	1775 (36.2)	502 (37.6)	
	Moderate	1005 (20.5)	260 (19.5)	
	Poor	274 (5.6)	69 (5.2)	
	Very poor	54 (1.1)	14 (1.0)	
Financial impact of COVID-19	Very high	1566 (27.8)	405 (26.3)	0.184
	High	916 (16.2)	227 (14.8)	
	Moderate	959 (17.0)	257 (16.7)	
	Low	810 (14.4)	248 (16.1)	
	None/very low	1387 (24.6)	401 (26.1)	
GHQ-28 mean score (SD)	Somatic symptoms	2.4 (2.1)	2.2 (2.1)	0.010
	Anxiety/Insomnia	3.2 (2.3)	2.9 (2.3)	<0.001
	Social dysfunction	2.2 (2.0)	2.1 (2.0)	0.21
	Severe depression	0.9 (1.5)	0.80 (1.4)	0.007
	Total score	8.7 (6.2)	8.1 (6.1)	0.001

GHQ = General Health Questionnaire; N = Number; SD = standard deviation;

^†^: Categorical data are compared with chi-square test, while numerical data are compared with one-way analysis of variance.

Of the 1538 women included in the final analyses (mean age: 35.1±12.2 years), a majority were professionally active (67.2%), with a university degree (82.6%). Only 15.8% of them had psychiatric history (depression: 9.2%; other disorder: 6.6%). Most of them were confined along with their families (47.4%) or a partner (26.9%), and reported excellent (36.8%) or good (37.6%) relationships with their companions during the lockdown. More than 4 in 10 respondents reported a high or very high negative impact of the COVID-19 lockdown on their financial situation, while only 1 in 4 were not negatively affected. More than 4 in 10 respondents reported a significant impact of the COVID-19 lockdown on their financial situation, while only 1 in 4 were unaffected.

Internal consistency estimates (Cronbach alphas) of GHQ-28 subscales were satisfactory in the present study, ranging from 0.74 for “social dysfunction” to 0.89 for “anxiety/insomnia”. Mean scores (±SD) were 2.2 ±2.1 for somatic symptoms; 2.9±2.3 for anxiety/insomnia; 2.1±2.0 for social dysfunction and 0.80±1.4 for severe depression. The GHQ-28 mean score went from 8.1±6.1 to 7.8±6.1 (*P* = 0.007) over the lockdown period. Detailed analyses (Table 2) indicate that subscales scores remained unchanged for most respondents, but increased for 6.0% and decreased for 7.1%.

**Table 2:** Changes in GHQ-28 subscales over the first lockdown period.

	N	Decrease	Stable	Increase
Subscales		N (%)	N (%)	N (%)
Somatic symptoms	1509	160 (10.6)	1258 (83.4)	91 (6.0)
Anxiety/Insomnia	1509	127 (8.4)	1254 (83.1)	128 (8.5)
Social dysfunction	1476	106 (7.2)	1268 (85.9)	102 (6.9)
Severe depression	1509	28 (1.9)	1438 (95.3)	43 (2.8)

GHQ = General Health Questionnaire. N = number; GHQ subscales scores were considered as having increased (or decreased) over the lockdown period if they have increased (or decreased) by at least 2 points between the first and the second assessment, and “unchanged” otherwise.

Physical and/or sexual assault was reported by 43.2% of women during lifetime and by 7.1% post lockdown (Table 3).

**Table 3:** Reported experiences of violence, by category (N = 1538).

	Lifetime	Post-lockdown
	N (%)	N (%)
Unwanted sexual contact	315 (20.5)	64 (4.2)
Physical assault	311 (20.2)	48 (3.1)
Sexual assault	294 (19.1)	38 (2.5)
Assault with weapon	71 (4.6)	10 (0.7)
At least one category	664 (43.2)	109 (7.1)

N = number.

In univariate analysis (Table 4), sexual and physical assault post lockdown was associated with status as a student, a history of abuse, and in those with increased somatic, anxiety and depression symptoms over the lockdown period. This risk decreased with age, higher satisfaction with relationships, and decreased social dysfunction GHQ-28 scores over the lockdown period (that is, these were protective factors). When these factors were input into multivariate analyses, the risk of violence after lockdown was associated with a history of abuse, and increased GHQ-28 anxiety scores over the lockdown period. The risk decreased with older age and decreased GHQ-28 social dysfunction scores over the lockdown period.

**Table 4:** Rate ratios and 95% confidence intervals (RR [95% CI]) of the number of reported experiences of violence/assault post first lockdown (N = 1538); Poisson regression.

		Univariate	Multivariate
Variables		RR [95% CI]	RR [95% CI]
Mean age in years (SD)		**0.96 [0.95–0.98]**	**0.97 [0.95–0.99]**
Professional status	Student	**1.51 [1.06–2.15]**	1.01 [0.62–1.65]
	Inactive	0.86 [0.50–1.45]	0.75 [0.40–1.40]
	Active	1	1
Education level	University degree	0.83 [0.39–1.79]	
	High school graduate	0.99 [0.43–2.29]	
	High school undergraduate	1	
Psychiatric history	Others disorders	1.20 [0.67–2.17]	
	Depression	1.44 [0.90–2.30]	
	None	1	
History of abuse		**4.53 [3.13–6.57]**	**4.94 [3.18–7.67]**
Lockdown Relationship status	With Friends/roommate	0.71 [0.37–1.37]	
	With Family	0.91 [0.56–1.46]	
	With a Partner	1.13 [0.69–1.87]	
	Alone	1	
Satisfaction with relationship (alone excluded)			
	Excellent	**0.25 [0.11–0.59]**	0.81 [0.26–2.52]
	Good	**0.20 [0.09–0.48]**	0.62 [0.20–1.92]
	Moderate	**0.21 [0.08–0.51]**	0.46 [0.15–1.48]
	Poor	0.41 [0.15–1.08]	0.97 [0.29–3.33]
	Very poor	1	
Financial impact of COVID-19	Very high	**1.66 [1.08–2.55]**	1.62 [0.97–2.71]
	High	1.40 [0.83–2.32]	1.70 [0.96–3.01]
	Moderate	1.18 [0.70–1.99]	1.01 [0.55–1.85]
	Low	1.04 [0.60–1.79]	1.03 [0.56–1.90]
	None/very low	1	1
Changes in GHQ-28 mean scores (SD) during lockdown			
Somatic symptoms	Increased	**1.70 [0.99–2.91]**	1.84 [0.95–3.56]
	Decreased	0.87 [0.52–1.45]	1.22 [0.66–2.23]
	Unchanged	1	1
Anxiety/Insomnia	Increased	**2.42 [1.55–3.77]**	**2.18 [1.28–3.72]**
	Decreased	1.26 [0.73–2.16]	**2.32 [1.25–4.30]**
	Unchanged	1	1
Social dysfunction	Increased	1.34 [0.75–2.38]	0.92 [0.47–1.77]
	Decreased	**0.36 [0.14–0.91]**	**0.29 [0.09–0.97]**
	Unchanged	1	1
Severe depression	Increased	**2.62 [1.45–4.73]**	1.20 [0.56–2.56]
	Decreased	0.59 [0.18–1.96]	1.20 [0.26–5.44]
	Unchanged	1	

GHQ = General Health Questionnaire; SD = standard deviation; RR = Rate Ratios; CI = Confidence Interval; significant results are marked in bold.

## Discussion

Psychological distress was elevated and remained stable for most of the 1538 female respondents over the lockdown period. Physical or sexual violence affected more than 7% of the women post-lockdown. Unwanted sexual contact accounted for the majority of violence, but physical and sexual assault were also prevalent. The risk of being assaulted increased with changes in anxiety/insomnia symptoms over the lockdown period, history of abuse. It decreased with older age and improved social dysfunction over the lockdown period.

Psychological distress was elevated in our study sample during the first lockdown, a result in line with others studies [26–29], mainly because of anxiety/insomnia, social dysfunction and somatization symptoms. However, depressive symptoms were minimal, and the mean GHQ-28 score slightly decreased at the end of the lockdown overall. This indicates that the psychological impact of the COVID-19 outbreak was significant on women participating in the study, but remained overall under control. This confirms previous findings showing that people in the French general population were well aware of the seriousness of the COVID-19, but had confidence in the recommended preventive measures to lessen the threat [4, 30]. Detailed analyses showed that a minority of participants experienced changes in psychological distress over the lockdown period, with almost equal proportions experiencing negative and positive changes. Post-hoc analyses showed that women with reduced scores at the end of the lockdown had higher psychological distress at baseline, and vice-versa.

While reduced well-being may result directly from lockdown restrictions and COVID-19 fear, observed improvements in mental state over the same period are more difficult to explain. One possible explanation is that the lockdown positively modified environmental and social factors accounting for psychological distress in a minority of women. Defining the exact nature of these changes warrants further investigation, but one study has shown some positive consequences of the lockdown, with 62% of participants reporting ’silver linings’, which included enjoying working from home, spending more time with family, and a quieter, less polluted environment [31]. Another study showed that spending time on leisure activities and spending time in-person with family and friends negatively correlated with psychological decline [32].

This relative stability of psychological distress during the first lockdown despite fear and restrictions, may be also related to our study sample characteristics, as it included a majority of women with privileged backgrounds. Most of them had a university degree, were professionally active, without psychiatric history, reported high levels of relationship satisfaction during the lockdown, and were only moderately impacted financially by the COVID-19 crisis. Nonetheless, more than 40% of them reported being assaulted during their lifetime, which is higher than in other studies [33, 34]. This may be because our estimate includes unwanted sexual contact, which is often overlooked in studies, despite having severe consequences for the victims [35]. Estimates in univariate analysis suggest that abuse history, younger age and increased psychological distress over the lockdown period are associated with an increased risk of violence post lockdown, together with several characteristics indicative of reduced social conditions, such as being a student [32], reporting very low satisfaction with lockdown companions, and severe financial difficulties related to COVID-19. This suggests that the likelihood of being assaulted post lockdown was associated with the negative psychological impact of the lockdown, but also possibly with vulnerability factors preexisting the COVID-19 pandemic.

In multivariate analysis, aggravated anxiety/insomnia symptoms remained associated with increased violence against women, confirming that reduced mental health increases the victimization risk [14]. This is likely because it makes women potentially more vulnerable to the perpetrators [36, 37]. However, improved anxiety/insomnia symptoms also became a significant predictor of abuse in multivariate analysis while deprivation indicators, in turn, became non-significant. This mediation effect could indicate that some women vulnerable to abuse actually improved their mental state during the lockdown, possibly in the absence of the perpetrators, but were exposed again afterwards. Contrary to other studies [38, 39], the risk of abuse was unrelated to marital/companionship status, but rather to social (dys)functioning. This confirms the protective influence of healthy social relationships towards violence against women, irrespective of relationship status [39–41].

This study must be interpreted considering its limitations. First, the important proportion of lost-to-follow-up respondents precluded the investigation of violence against women in the total study sample. This is all the more concerning that women lost to follow-up seemed more vulnerable than others to psychological distress during lockdown. In addition, recruitment using social media tends to lead to an over-representation of younger participants with higher education and income [42, 43], which may limit the generalizability of our results, as these participants may have been relatively protected from the negative social impacts of the COVID-19 lockdown. Finally, some major vulnerability factors to violence against women, such as drug or alcohol misuse, were not assessed. The absence of baseline assessment prior to lockdown, and response bias in self-reported data also constitute limitations to the study design. Since the monitoring of lockdown adverse effects is still suboptimal, the large size of our cohort and the prospective study design nevertheless has offered a unique opportunity to investigate violence against women amid the COVID-19 pandemic.

## Conclusion

Women who experienced changes in anxiety/insomnia symptoms during the COVID-19 lockdown were at higher risk than others of being abused in its aftermath, especially when they were already socially vulnerable. While social and psychological factors accounting for these changes warrant further investigation, communication and preventive measures during pandemics should hitherto include initiatives towards women vulnerable to abuse.

## References

[1] ECDC. COVID-19 situation update worldwide, as of 2 July 2020. Stockholm: European Centre for Disease Prevention and Control 2020.

[2] Stanton R, To QG, Khalesi S, Williams SL, Alley SJ, Thwaite TL, et al. Depression, Anxiety and Stress during COVID-19: Associations with Changes in Physical Activity, Sleep, Tobacco and Alcohol Use in Australian Adults. Int J Environ Res Public Health. 2020;17(11). Epub 2020/06/11. doi: 10.3390/ijerph17114065. 32517294 PMC7312903

[3] Brooks SK, Webster RK, Smith LE, Woodland L, Wessely S, Greenberg N, et al. The psychological impact of quarantine and how to reduce it: rapid review of the evidence. Lancet. 2020;395(10227):912–20. Epub 2020/03/01. doi: 10.1016/S0140-6736(20)30460-8 . 32112714 PMC7158942

[4] Constant A, Conserve DF, Gallopel-Morvan K, Raude J. Socio-Cognitive Factors Associated With Lifestyle Changes in Response to the COVID-19 Epidemic in the General Population: Results From a Cross-Sectional Study in France. Front Psychol. 2020;11:579460. Epub 2020/11/03. doi: 10.3389/fpsyg.2020.579460. 33132989 PMC7550454

[5] WHO. Mental health and psychosocial considerations during the COVID-19 outbreak, 18 March 2020. Geneva: World Health Organization, 2020 2020. Report No.: Contract No.: WHO/2019-nCoV/MentalHealth/2020.1.

[6] Salari N, Khazaie H, Hosseinian-Far A, Khaledi-Paveh B, Kazeminia M, Mohammadi M, et al. The prevalence of stress, anxiety and depression within front-line healthcare workers caring for COVID-19 patients: a systematic review and meta-regression. Hum Resour Health. 2020;18(1):100. Epub 2020/12/19. doi: 10.1186/s12960-020-00544-1. 33334335 PMC7745176

[7] Olashore AA, Akanni OO, Fela-Thomas AL, K. K. The psychological impact of COVID-19 on health-care workers in African Countries: A systematic review. Asian J Soc Health Behav 2021;4:85–97.

[8] Sharma R, Bansal P, Chhabra M, Bansal C, Arora M. Severe acute respiratory syndrome coronavirus-2-associated perceived stress and anxiety among indian medical students: A cross-sectional study. Asian J Soc Health Behav. 2021;4:98–104.

[9] Rajabimajd N, Alimoradi Z, Griffiths M. Impact of COVID-19-related fear and anxiety on job attributes: A systematic review. Asian J Soc Health Behav. 2021;4:51–5.

[10] Salari N, Hosseinian-Far A, Jalali R, Vaisi-Raygani A, Rasoulpoor S, Mohammadi M, et al. Prevalence of stress, anxiety, depression among the general population during the COVID-19 pandemic: a systematic review and meta-analysis. Global Health. 2020;16(1):57. Epub 2020/07/08. doi: 10.1186/s12992-020-00589-w. 32631403 PMC7338126

[11] Cenat JM, Blais-Rochette C, Kokou-Kpolou CK, Noorishad PG, Mukunzi JN, McIntee SE, et al. Prevalence of symptoms of depression, anxiety, insomnia, posttraumatic stress disorder, and psychological distress among populations affected by the COVID-19 pandemic: A systematic review and meta-analysis. Psychiatry Res. 2020;295:113599. Epub 2020/12/08. doi: 10.1016/j.psychres.2020.113599. 33285346 PMC7689353

[12] Shah SMA, Mohammad D, Qureshi MFH, Abbas MZ, Aleem S. Prevalence, Psychological Responses and Associated Correlates of Depression, Anxiety and Stress in a Global Population, During the Coronavirus Disease (COVID-19) Pandemic. Community Ment Health J. 2020. Epub 2020/10/28. doi: 10.1007/s10597-020-00728-y. 33108569 PMC7590908

[13] Zhang Y, Wang S, Ding W, Meng Y, Hu H, Liu Z, et al. Status and influential factors of anxiety depression and insomnia symptoms in the work resumption period of COVID-19 epidemic: A multicenter cross-sectional study. J Psychosom Res. 2020;138:110253. Epub 2020/09/27. doi: 10.1016/j.jpsychores.2020.110253. 32979696 PMC7500335

[14] Dillon G, Hussain R, Loxton D, Rahman S. Mental and Physical Health and Intimate Partner Violence against Women: A Review of the Literature. Int J Family Med. 2013;2013:313909. Epub 2013/02/23. doi: 10.1155/2013/313909. 23431441 PMC3566605

[15] Bacchus LJ, Ranganathan M, Watts C, Devries K. Recent intimate partner violence against women and health: a systematic review and meta-analysis of cohort studies. BMJ Open. 2018;8(7):e019995. Epub 2018/07/30. doi: 10.1136/bmjopen-2017-019995. 30056376 PMC6067339

[16] Mazza M, Marano G, Lai C, Janiri L, Sani G. Danger in danger: Interpersonal violence during COVID-19 quarantine. Psychiatry Res. 2020;289:113046. Epub 2020/05/11. doi: 10.1016/j.psychres.2020.113046. 32387794 PMC7190494

[17] Gosangi B, Park H, Thomas R, Gujrathi R, Bay CP, Raja AS, et al. Exacerbation of Physical Intimate Partner Violence during COVID-19 Lockdown. Radiology. 2020:202866. Epub 2020/08/14. 10.1148/radiol.2020202866 PMC7427119 32787700

[18] Evans DP. COVID-19 and violence: a research call to action. BMC Womens Health. 2020;20(1):249. Epub 2020/11/12. doi: 10.1186/s12905-020-01115-1. 33172466 PMC7653443

[19] Viero A, Barbara G, Montisci M, Kustermann K, Cattaneo C. Violence against women in the Covid-19 pandemic: A review of the literature and a call for shared strategies to tackle health and social emergencies. Forensic Sci Int. 2020;319:110650. Epub 2020/12/20. doi: 10.1016/j.forsciint.2020.110650. 33340849 PMC8021946

[20] Ashraf A, Ali I, Ullah F. Domestic and gender-Based violence: Pakistan scenario amidst COVID-19. Asian J Soc Health Behav 2021;4:47.

[21] Humphreys KL, Myint MT, Zeanah CH. Increased Risk for Family Violence During the COVID-19 Pandemic. Pediatrics. 2020;146(1). Epub 2020/04/23. doi: 10.1542/peds.2020-0982. 32317306

[22] Campbell AM. An increasing risk of family violence during the Covid-19 pandemic: Strengthening community collaborations to save lives. Forensic Science International Reports. 2020;2:100089-. Epub 2020/04/12. doi: 10.1016/j.fsir.2020.100089 PMC7152912 38620174

[23] de Mont-Marin F, Hardy P, Lepine JP, Halfon P, Feline A. [Validation of a French version of the General Health Questionnaire (GHQ-28) in a diabetic population]. Encephale. 1993;19(4):293–301. Epub 1993/07/01. . 8275916

[24] Pariente PD, Challita H, Mesbah M, Guelfi JD. The GHQ-28 questionnaire in French: a validation survey in a panel of 158 general psychiatric patients. European Psychiatry. 1992;7(1):15–20. Epub 2020/04/16. doi: 10.1017/S0924933800002455

[25] Chiasson M, Lapierre S, Balbinotti MAA, Desjardins S, Vasiliadis HM. Validation de contenu de la version francophone du questionnaire Impact of Event Scale-Revised selon les critères du DSM-5. Pratiques Psychologiques. 2018;24(1):21–34. 10.1016/j.prps.2017.02.002.

[26] Lal A, Sanaullah A, MK MS, Ahmed N, Maqsood A, Ahmed N. Psychological Distress among Adults in Home Confinement in the Midst of COVID-19 Outbreak. Eur J Dent. 2020. Epub 2020/11/27. doi: 10.1055/s-0040-1718644. 33242914 PMC7840433

[27] Odriozola-Gonzalez P, Planchuelo-Gomez A, Irurtia MJ, de Luis-Garcia R. Psychological symptoms of the outbreak of the COVID-19 confinement in Spain. J Health Psychol. 2020:1359105320967086. Epub 2020/10/31. doi: 10.1177/1359105320967086 33124471

[28] Husky MM, Kovess-Masfety V, Swendsen JD. Stress and anxiety among university students in France during Covid-19 mandatory confinement. Compr Psychiatry. 2020;102:152191. Epub 2020/07/21. doi: 10.1016/j.comppsych.2020.152191. 32688023 PMC7354849

[29] Bartoszek A, Walkowiak D, Bartoszek A, Kardas G. Mental Well-Being (Depression, Loneliness, Insomnia, Daily Life Fatigue) during COVID-19 Related Home-Confinement-A Study from Poland. Int J Environ Res Public Health. 2020;17(20). Epub 2020/10/16. doi: 10.3390/ijerph17207417. 33053787 PMC7599953

[30] Peretti-Watel P, Verger P, Launay O, Group CS. The French general population’s attitudes toward lockdown against COVID-19: a fragile consensus. BMC Public Health. 2020;20(1):1920. Epub 2020/12/20. doi: 10.1186/s12889-020-10048-1. 33339543 PMC7746918

[31] Every-Palmer S, Jenkins M, Gendall P, Hoek J, Beaglehole B, Bell C, et al. Psychological distress, anxiety, family violence, suicidality, and wellbeing in New Zealand during the COVID-19 lockdown: A cross-sectional study. PLoS One. 2020;15(11):e0241658. Epub 2020/11/05. doi: 10.1371/journal.pone.0241658. 33147259 PMC7641386

[32] Ryerson NC. Behavioral and Psychological Correlates of Well-Being during COVID-19. Psychol Rep. 2020:33294120978160. Epub 2020/12/12. doi: 10.1177/0033294120978160. 33302799

[33] Sanz-Barbero B, Lopez Pereira P, Barrio G, Vives-Cases C. Intimate partner violence against young women: prevalence and associated factors in Europe. J Epidemiol Community Health. 2018;72(7):611–6. Epub 2018/03/10. doi: 10.1136/jech-2017-209701 . 29519883

[34] World Health O. Global and regional estimates of violence against women: prevalence and health effects of intimate partner violence and non-partner sexual violence. Geneva: World Health Organization; 2013 2013.

[35] Stahlman S, Javanbakht M, Cochran S, Hamilton AB, Shoptaw S, Gorbach PM. Mental Health and Substance Use Factors Associated With Unwanted Sexual Contact Among U.S. Active Duty Service Women. J Trauma Stress. 2015;28(3):167–73. Epub 2015/05/16. doi: 10.1002/jts.22009 . 25976935 PMC4522288

[36] Scholle SH, Rost KM, Golding JM. Physical abuse among depressed women. J Gen Intern Med. 1998;13(9):607–13. Epub 1998/10/01. doi: 10.1046/j.1525-1497.1998.00183.x . 9754516 PMC1497013

[37] Trevillion K, Oram S, Feder G, Howard LM. Experiences of domestic violence and mental disorders: a systematic review and meta-analysis. PLoS One. 2012;7(12):e51740. Epub 2013/01/10. doi: 10.1371/journal.pone.0051740. 23300562 PMC3530507

[38] Flury M, Nyberg E, Riecher-Rossler A. Domestic violence against women: Definitions, epidemiology, risk factors and consequences. Swiss Med Wkly. 2010;140:w13099. Epub 2010/09/21. doi: 10.4414/smw.2010.13099. 20853195

[39] Hellmann DF, Kinninger MW, Kliem S. Sexual Violence against Women in Germany: Prevalence and Risk Markers. Int J Environ Res Public Health. 2018;15(8). Epub 2018/08/01. doi: 10.3390/ijerph15081613. 30061527 PMC6121316

[40] Yakubovich AR, Stockl H, Murray J, Melendez-Torres GJ, Steinert JI, Glavin CEY, et al. Risk and Protective Factors for Intimate Partner Violence Against Women: Systematic Review and Meta-analyses of Prospective-Longitudinal Studies. Am J Public Health. 2018;108(7):e1–e11. Epub 2018/05/18. doi: 10.2105/AJPH.2018.304428 . 29771615 PMC5993370

[41] Williamson HC. Early Effects of the COVID-19 Pandemic on Relationship Satisfaction and Attributions. Psychol Sci. 2020;31(12):1479–87. Epub 2020/11/06. doi: 10.1177/0956797620972688 . 33151125 PMC7797601

[42] Whitaker C, Stevelink S, Fear N. The Use of Facebook in Recruiting Participants for Health Research Purposes: A Systematic Review. J Med Internet Res. 2017;19(8):e290. Epub 2017/08/31. doi: 10.2196/jmir.7071. 28851679 PMC5594255

[43] Topolovec-Vranic J, Natarajan K. The Use of Social Media in Recruitment for Medical Research Studies: A Scoping Review. Journal of medical Internet research. 2016;18(11):e286–e. doi: 10.2196/jmir.5698 . 27821383 PMC5118584

